# Comparing Imputation Procedures for Affymetrix Gene Expression Datasets Using MAQC Datasets

**DOI:** 10.1155/2013/790567

**Published:** 2013-10-09

**Authors:** Sreevidya Sadananda Sadasiva Rao, Lori A. Shepherd, Andrew E. Bruno, Song Liu, Jeffrey C. Miecznikowski

**Affiliations:** ^1^Department of Biostatistics, Roswell Park Cancer Institute, Buffalo, NY 14263, USA; ^2^Center for Computational Research, University at Buffalo, NYS Center of Excellence in Bioinformatics and Life Sciences, Buffalo, NY 14203, USA; ^3^Department of Biostatistics, SUNY University at Buffalo, Buffalo, NY 14214, USA

## Abstract

*Introduction*. The microarray datasets from the MicroArray Quality Control (MAQC) project have enabled the assessment of the precision, comparability of microarrays, and other various microarray analysis methods. However, to date no studies that we are aware of have reported the performance of missing value imputation schemes on the MAQC datasets. In this study, we use the MAQC Affymetrix datasets to evaluate several imputation procedures in Affymetrix microarrays. *Results.* We evaluated several cutting edge imputation procedures and compared them using different error measures. We randomly deleted 5% and 10% of the data and imputed the missing values using imputation tests. We performed 1000 simulations and averaged the results. The results for both 5% and 10% deletion are similar. Among the imputation methods, we observe the local least squares method with *k* = 4 is most accurate under the error measures considered. The *k*-nearest neighbor method with *k* = 1 has the highest error rate among imputation methods and error measures. *Conclusions.* We conclude for imputing missing values in Affymetrix microarray datasets, using the MAS 5.0 preprocessing scheme, the local least squares method with *k* = 4 has the best overall performance and *k*-nearest neighbor method with *k* = 1 has the worst overall performance. These results hold true for both 5% and 10% missing values.

## 1. Introduction

In microarray experiments, randomly missing values may occur due to scratches on the chip, spotting errors, dust, or hybridization errors. Other nonrandom missing values may be biological in nature, for example, probes with low intensity values or intensity values that may exceed a readable threshold. These missing values will create incomplete gene expression matrices where the rows refer to genes and the columns refer to samples. These incomplete expression matrices will make it difficult for researchers to perform downstream analyses such as differential expression inference, clustering or dimension reduction methods (e.g., principal components analysis), or multidimensional scaling. Hence, it is critical to understand the nature of the missing values and to choose an accurate method to impute the missing values. 

There have been several methods put forth to impute missing data in microarray experiments. In one of the first papers related to microarrays, Troyanskaya et al. [1] examine several methods of imputing missing data and ultimately suggest a *k*-nearest neighbors approach. Researchers also explored applying previously developed schemes for microarrays such as the nonlinear iterative partial least squares (NIPALS) as discussed by Wold [2]. A Bayesian approach for missing data in gene expression microarrays is provided by Oba et al. [3]. Other approaches such as that of Bø et al. [4] suggest using least squares methods to estimate the missing values in microarray data, while Kim et al. [5] suggest using a local least squares imputation. A Gaussian mixture method for imputing missing data is proposed by Ouyang et al. [6].

While many of these approaches can be generally applied to different types of gene expression arrays, we will focus on applying these methods to Affymetrix gene expression arrays, one of the most popular arrays in scientific research. Naturally, when proposing a new imputation scheme for expression arrays, it is necessary to compare the new method against existing methods. Several excellent papers have compared missing data procedures on high throughput data platforms such as in two-dimensional gel electrophoresis as in Miecznikowski et al.'s works [7] or gene expression arrays [8–10]. Before studying missing data imputation schemes in Affymetrix gene expression arrays, it is reasonable to first remove any existing missing values. In this way, we ensure that any subsequent missing values have known true values. A detection call algorithm is used to filter and remove missing expression values based on absent/present calls [11]. Subsequently, a preprocessing scheme is then employed. There are numerous tasks to perform in preprocessing Affymetrix arrays, including background adjustment, normalization, and summarization. A good overview of the methods available for preprocessing is provided by Gentleman et al. [12]. For our analysis, the detection call employs MAS 5.0 [13] to obtain expression values; thus, we also use the MAS 5.0 suite of functions as our preprocessing method. 

For our analysis, we focus on the microarray quality control (MAQC) datasets (Accession no. GSE5350), where the datasets have been specifically designed to address the points of strength and weakness of various microarray analysis methods. The MAQC datasets were designed by the US Food and Drug Administration to provide quality control (QC) tools to the microarray community to avoid procedural failures. The project aimed to develop guidelines for microarray data analysis by providing the public with large reference datasets along with readily accessible reference ribonucleic acid (RNA) samples. Another purpose of this project was to establish QC metrics and thresholds for objectively assessing the performance achievable by various microarray platforms. These datasets were designed to evaluate the advantages and disadvantages of various data analysis methods. 

The initial results from the MAQC project were published in Shi's work [14] and later in Chen et al.'s work [15] and Shi et al.'s work [16]. Specifically, the MAQC experimental design for Affymetrix gene expression HG-U133 Plus 2.0 GeneChip includes 6 different test sites, 4 pools per site, and 5 replicates per site, for a total of 120 arrays (see Section 2). This rich dataset provides an ideal setting for evaluating imputation methods on Affymetrix expression arrays. While this dataset has been mined to determine inter-intra platform reproducibility of measurements, to our knowledge, none has studied imputation methods on this dataset. 

The MAQC dataset hybridizes two RNA sample types—Universal Human Reference RNA (UHRR) from Stratagene and a Human Brain Reference RNA (HBRR) from Ambion. These 2 reference samples and varying mixtures of these samples constitute the 4 different pools included in the MAQC dataset. By using various mixtures of UHRR and HBRR, this dataset is designed to study technical variations present in this technology. By technical variations, we are referring to the variability between preparations and labeling of sample, variability between hybridization of the same sample to different arrays, testing site variability, and variability between the signal on replicate features of the same array. Meanwhile, biological variability refers to variability between individuals in population and is independent of the microarray process itself. By the MAQC dataset being designed to study technical variation, we can examine the accuracy of the imputation procedures without the confounding feature of biological variability. Other than MAQC datasets, similar technical datasets have been used to evaluate different analysis methods specific to Affymetrix microarrays, for example, methods for identifying differentially expressed genes [17–19]. 

In summary, our analysis examines cutting edge imputation schemes on an Affymetrix technical dataset with minimal biological variation. Section 2 discusses the MAQC dataset and the proposed imputation schemes. Meanwhile, Section 3 describes the results from applying the imputation methods for addressing missingness in the MAQC datasets. Finally, we conclude our paper with a discussion and conclusion in Sections 4 and 5. 

## 2. Materials and Methods

### 2.1. Datasets

The MAQC experiments and datasets are fully described by Shi [14]. The MAQC dataset hybridizes 2 RNA samples a Universal Human Reference RNA (UHRR) from Stratagene and a Human Brain Reference RNA (HBRR) from Ambion. From these 2 samples, 4 pools are created, that is, the 2 reference RNA samples as well as 2 mixtures of the original samples: Pool A, 100% UHRR; Pool B, 100% HBRR; Pool C, 75% UHRR and 25% HBRR; and Pool D, 25% UHRR and 75% HBRR. Both Pool A and Pool B are commercially available and biologically distinct where we expect a large number of differentially expressed genes between Pool A and Pool B. 

There are 6 different test sites where each test site assayed the 4 pools with 5 replicates per pool. Thus, for each test site there are a total of 20 arrays and thus a total of 120 arrays over the 6 sites. The data is examined separately for each pool (4) and each site (6) separately yielding 24 “site and pool datasets.” 

### 2.2. Missing Values and Detection Call Algorithm

Using MAS 5.0, a detection call algorithm is used to flag the missing values [13]. The detection call determines if the transcript of a gene is present or absent in the sample. For every gene, the microarray chip has probes that perfectly match a segment of the gene sequence (PM probes) and probes that contain a single mismatched nucleotide in the center of the perfect match probe (MM probes). The difference in the intensity of the perfect and mismatch probes is used to make detection calls. 

The detection call algorithm is further summarized by Mei et al. [11]. For each genetic transcript, there is a probe set with 11 to 20 probe pairs where a probe pair consists of a PM probe and MM probe. In short, discrimination scores are calculated for each probe set from the raw intensity data for the probe pairs in the probe set. For each probe pair, the ratio of the sum and difference of the PM and MM probes gives the discrimination score for that probe pair. This score is calculated for all the probe pairs in a probe set. The null hypothesis is that the median discrimination score of a probe set is equal to *τ*, and the alternate hypothesis is that the median discrimination score is greater than *τ*, where *τ* is defined as a small nonnegative number which can be changed by the user to adjust the specificity and sensitivity. One-sided Wilcoxon rank sum tests are performed for each probe set. Two significance levels *α*_1_ and *α*_2_, act as the cutoffs for the *P* values for probe set detection calls. A present call is made for a probe set (transcript) with a *P* value <*α*_1_, an absent call for a transcript with *P* value ≥*α*_2_ and a marginally detected call for a transcript with *α*_1_ ≤ *P*  value < *α*_2_. We use the MAS 5.0 preset values 0.04, 0.06, and 0.015 for *α*_1_, *α*_2_, *τ*, respectively, to determine if the probe set is present, marginally present, or absent in the sample. 

### 2.3. Percent Present Algorithm

We use the “mas5calls” function detailed in Affymetrix [20] from the “affy” package [13] to make the detection calls. Using this function, we get a present, marginal, or absent call for each probe set in each array. For every sample, probe sets were filtered based on the present calls where probe sets that were present in all 5 replicates of a given pool and a given site were retained for further analyses. Probe sets that were detected as absent or marginally present in 1 or more replicates of a sample were removed. This creates a complete expression matrix for each site and pool combination. 

The SimpleAffy R package has methods for quality control metrics on Affymetrix arrays [21]. One metric is percent present call which calculates the percentage of present probe sets in each array. Using this metric, we calculate the percent present calls for all 120 arrays separately and then average the percentages over the 5 replicates for each sample and each site. 

### 2.4. Preprocessing Algorithm

 We pre-process each complete expression matrix using MAS 5.0 available in Bioconductor [22] to obtain expression values for further analyses. The MAS 5.0 preprocessing was implemented using the R language “affy” library [13]. 

Preprocessing algorithms for Affymetrix gene expression microarrays are necessary to account for the systematic variation present in array technology and to summarize the signal for each gene which is measured via a series of probe sets. As discussed by Gentleman et al. [12], preprocessing schemes can be organized into three steps: a background adjustment step, a normalization step, and a summarization step. In short, the MAS 5.0 preprocessing algorithm is outlined in the Statistical Algorithms Description Document [20] and used in the MAS 5.0 software Affymetrix [20]. The steps in MAS 5.0 involve (1) a weighted nearest neighbor step to estimate and remove the background signal, (2) a normalization step that scales all arrays to a baseline array, and (3) a summarization step using an ideal mismatch, which may be slightly different than the perfect mismatch probe described earlier. 

To compare imputation methods, we randomly remove a percentage of the probe set expression values from the complete expression matrix and compare the complete dataset and the dataset(s) with the missing probe sets expression values estimated via an imputation method. We randomly remove 5% and 10% of the probe set expression values from the complete expression matrix with 1000 Monte-Carlo simulations at each deletion percentage. 

### 2.5. Missing Value Imputation Methods

Similar to the analysis by Oh et al. [10], we examine the following missing data analysis methods for the MAQC dataset:

1. row average (ROW),
2. *k* nearest neighbors using Euclidean distance or Pearson correlation, with *k* = 1 or 5, where *k* is the number of neighbors in the imputation (KNN),
3. singular value decomposition (SVD) [1],
4. least squares adaptive (LSA) [4],
5. local least squares (LLS), choosing *k* = 1, 3, and 4, where *k* is the cluster size used for regression [5],
6. Bayesian principal components analysis (BPCA) [3], and
7. noniterative partial least squares (NIPALS) [2].

Note that the row average method (ROW) and *k*-nearest neighbor (KNN) imputation were done using the R computing language with the *impute* package [23] while LSA was implemented using the Java language code [24]. In the ROW method, the average of the values that are present for that particular probe set is used to replace the missing probe set expression values. The KNN algorithm classifies objects based on closest (“nearest”) probe sets. In this algorithm, we find the *k*-nearest neighbors using a suitable distance metric, and then we impute the missing elements by averaging those (nonmissing) values of its neighbors. In the KNN method, there are different types of distance metrics (Pearson correlation, Euclidean, Mahalanobis, and Chebyshev distance) that can be employed. We chose the Euclidean distance metric as it has been reported to be more accurate [25]. 

Singular value decomposition (SVD) reduces the dimension of the data matrix and uses the global information in the data matrix to predict the missing values as detailed by Troyanskaya et al. [1]. Initially, all missing values are replaced by the row average. With this complete gene matrix, SVD is used to obtain “eigen genes” which are the orthogonal principal components. Then, the nonmissing values are regressed against the most significant eigen genes, and the regression function is used to predict the missing values. Using an expectation-maximization algorithm, missing values are estimated repeatedly until the total change in the expression matrix falls below the empirically determined threshold of 0.01.

The least squares adaptive method (LSA) is a combination of gene-based and array-based estimates of the missing values. The gene-based estimate is based on the correlation between genes and the array-based estimate is based on the correlation between arrays. A weighted average of these two estimates predicts the missing value. The weight is chosen to minimize the sum of squared errors for the new estimate. An adaptive weighting procedure is used which takes into account the strength of the gene correlation in the gene-based estimates. The LSA method is fully described by Bø et al. [4] and was implemented using the LSimpute.jar java code available at http://www.ii.uib.no/_trondb/imputation/.

The LLS method is a neighbor-based approach that selects neighbors based on their Pearson correlation coefficient. Multiple regression is performed using *k*-nearest neighbors as described by Kim et al. [5], and the LLS method is implemented using the R package “pcaMethods” [26]. The method restricts *k* to be less than the number of replicates/columns. In our case, with 5 replicates, we chose *k* equal to 1, 3, or 4. Global based methods, SVD [1] and BPCA [3], were implemented using the R package pcaMethods [26]. The NIPALS method is summarized by Wold [2] and is implemented using the R package “pcaMethods” [26]. Similar to KNN, in order to implement the NIPALS algorithm, it is necessary for the user to specify the number of principal components. To evaluate the different methods of imputation, probe set expression values were randomly deleted from the complete dataset, and the summary measures in the next section were compared across the methods. 

### 2.6. Quantitative Error Evaluation

The complete expression matrices for each pool and site are such that the rows correspond to probe sets, and the columns correspond to samples. Similar to Oh et al. [10], we denote this complete expression matrix as CD = (*y*_*gs*_)_*G*×*S*_, where *y*_*gs*_ is the expression intensity of probe set (roughly speaking “gene”) *g* on sample *s*. To simulate the missing data, we randomly remove 5% or 10% of the entries in CD. Then given a missing value imputation scheme, the missing value for probe set *g*, sample *s*, is imputed as ${\hat{y}}_{gs}$ and the imputed dataset is denoted as ID. 

To compare the imputed dataset ID with the complete dataset CD, we employ the following summary statistics:

(1) root mean squared error (RMSE),
  (1) RMSE=1  no.  of  missing  ∑{ygs  missing}(y^gs−ygs)2,
(2) relative estimation error (RAE) [25],
  (2) RAE=1  no.  of  missing  ∑{ygs  missing}|y^gs−ygs|ϕ(ygs),
 where
  (3) ϕ(ygs)={|ygs|,  if  |ygs|>ϵ,ϵ,  if  |ygs|<ϵ,
 (3) logged RMSE (LRMSE) [8],
  (4) LRMSE=1  no.  of  missing  ∑{xgs  missing}(x^gs−xgs)2,
 where ${\hat{x}}_{gs}=\log\;\left({\hat{y}}_{gs}\right)$, and
(4) RAE-L2 [10],
  (5) RAE-L2=1  no.  of  missing  ∑{ygs  missing}(y^gs−ygs)2ygs.

See Section 4 for the motivation for using these error measures to evaluate the imputation methods. To understand the variability in the imputation procedures, we perform each missing data simulation 1000 times.

### 2.7. Ranking the Imputation Methods

To identify the overall best and worst performing imputation methods (IM), we rank the IM based on their average performance across the different error measures, all pools, and all sites. The ranking procedure is carried out separately for 5% and 10% deletion. 

For each simulation, we compute 4 error measures for each of the 10 imputation methods. Averaging over the 1000 simulations, we get an average error value for each imputation method for every site and pool combination. For example, for the metric RMSE, there are 10 values: 1 for each imputation method at, say, Site 1 and Pool A. 

Then, we rank the 10 IM based on each error measure separately for each site and pool combination. For example, based on RMSE values, the IM are ranked from the lowest to highest; the IM with lowest RMSE value is 1 and the IM with the highest RMSE is 10. The IM each have a rank value for a given error measure at each site for each of the 4 pools. 

For every imputation method, the error measure rank values are averaged across the 6 sites for each pool; thus we obtain 4 average rank values, 1 for each pool. Finally, we average these 4 rank values to obtain a single number that gives a global ranking to every imputation method, reflecting its overall performance across different error measures, sites, and pools for a given deletion percentage. 

## 3. Results

We summarize our findings in two ways: probe set detection call summaries and error metrics and rankings for IM. Detection call results compare sites and pools while IM results choose the best imputation method based on the error metrics discussed in Section 2.

### 3.1. Detection Call Algorithm Results

**Figure 1:**
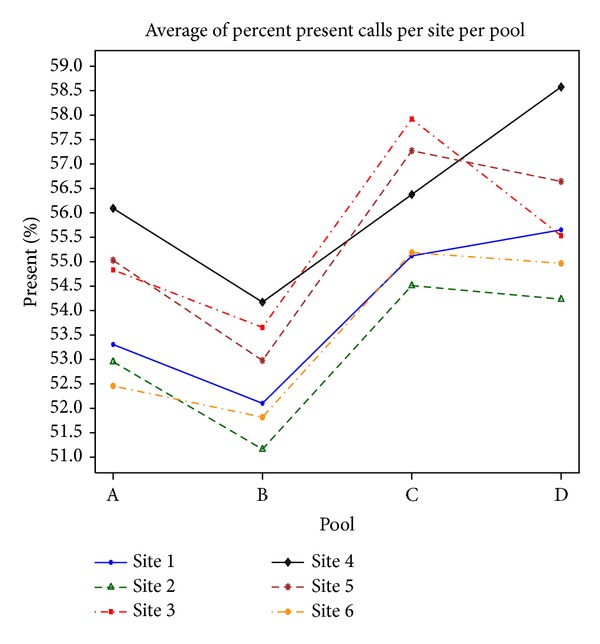
Percent present across pools and sites. Each curve shows a different site, and the *x*-axis shows the 4 pools and the *y*-axis shows the mean percentage of present probes on the Affymetrix arrays. Pool B has the smallest percentage of present probes, while Pool D has the largest percentage of present probes. Site 4 has the highest percentage of present probes, while Site 2 has the lowest percentage of present probes.

Across the 120 samples, as shown in Figure 1 the percent present calls has a minimum value of 51% and a maximum value of 58.5%. We observe that Site 4 have the highest mean percent present calls and Site 2 has the lowest mean percent present calls for probe sets. In terms of pools, Pool B has the lowest mean percent present calls for probe sets while Pool D has the highest mean percent present calls (see Figure 1). We performed an analysis of variance (ANOVA) to examine the effects of site and pool on the percentage of present probe sets in a microarray. The *P* values for site and pool are <0.0001 indicating significant site and pool effects. Nevertheless, the smallest percent present is 49.77 while the largest percent present is 63.69. These results indicate that the percentage of present probes is sensitive to site and pool and could be caused by the wet lab preparation of each pool and/or slight differences in each laboratory's (site) microarray protocols. Regardless of these subtle differences, we believe that percent present calls are similar across sites and pools, and hence it is reasonable to compare the subsequent IM results across the different sites and pools. 

The Affymetrix HG-U133 chip has 54675 probe sets. After filtering the absent calls, the number of present probe sets ranges from 22,900 (Pool B, Site 2) to 27,021 (Pool C, Site 3). The number of present probe sets for Site 1 is 24,184 (Pool A), 23,557 (Pool B), 25,163 (Pool C), and 25,318 (Pool D). Further tables and graphs representing the percent present calls and present probe sets for each pool and site can be found in Sadasiva Rao et al.'s work [27]. 

### 3.2. Imputation Results

The imputation methods are ranked based on average rank performance as described in Section 2, and the results are summarized in Table 1. Based on this ranking, the results are very similar at both 5% and 10% deletion. RMSE metric suggests that LSA imputation method has the best performance. With LRMSE and RAEL2 metrics, ROW is the best imputation method. The imputation method LLS with *k* = 4 has the best performance with the RAE (*ϵ* = 0.20) metric. 

From Table 1, we observe that KNN with *k* = 1 has the highest value for any given error measure; thus, it is the worst performing imputation method across all pools and sites. LLS with *k* = 4 has the overall best performance across the different error measures. 

**Figure 2:**
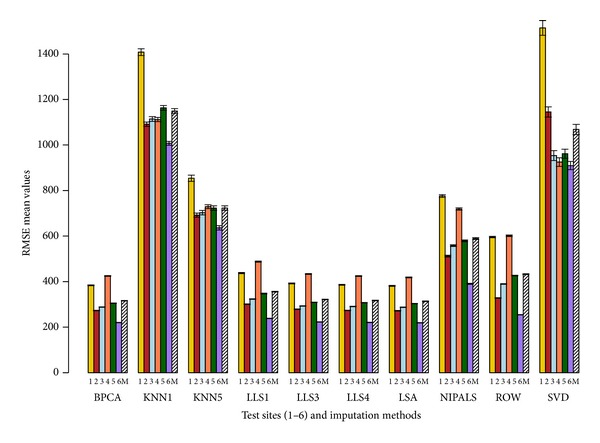
Average RMSE barplot with error bars. RMSE values are represented on the *y*-axis. The *x*-axis has the 6 sites (1, 2, 3, 4, 5, and 6) and 10 imputation tests (BPCA, KNN with *k* = 1,5, LLS with *k* = 1,3, 4, LSA, NIPALS, ROW, and SVD). Mean (M) depicted by the slashed bar is the overall mean for individual IM where the RMSE values are averaged across the 4 pools and 6 sites. This figure shows the performance of the 10 imputation tests using the RMSE metric with 5% deletion of values. 1000 simulations were performed where each simulation generated a dataset containing 5% missing values by randomly removing probe set values from the complete expression matrix of probe sets. Missing values were imputed using the 10 imputation tests. The results are compared using the RMSE metric (see Section 2). The RMSE values are averaged across the 4 pools. LSA has the best performance as it has the lowest RMSE value for a given site. KNN with *k* = 1 has the highest RMSE value and has the worst performance for all pools and all sites.

**Figure 3:**
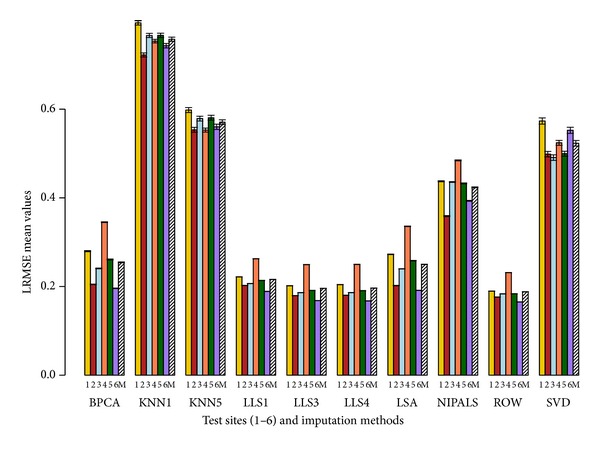
Average LRMSE barplot with error bars. LRMSE values are represented on the *y*-axis. The *x*-axis has the 6 sites (1, 2, 3, 4, 5, and 6) and 10 imputation tests (BPCA, KNN with *k* = 1,5, LLS with *k* = 1,3, 4, LSA, NIPALS, ROW, and SVD). Mean (M) depicted by the slashed bar represents the overall mean for individual IM where the LRMSE values are averaged across the 4 pools and 6 sites. This figure shows the performance of the 10 imputation tests using the RMSE metric with 5% deletion of values. 1000 simulations were performed where each simulation generated a dataset containing 5% missing values by randomly removing probe set values from the complete expression matrix of probe sets. Missing values were imputed using the 10 imputation tests. The results are compared using the LRMSE metric (see Section 2). The LRMSE values are averaged across the 4 pools. ROW has the best performance as it has the lowest LRMSE value for a given site. KNN with *k* = 1 has the highest LRMSE value and has the worst performance for all pools and all sites.

**Figure 4:**
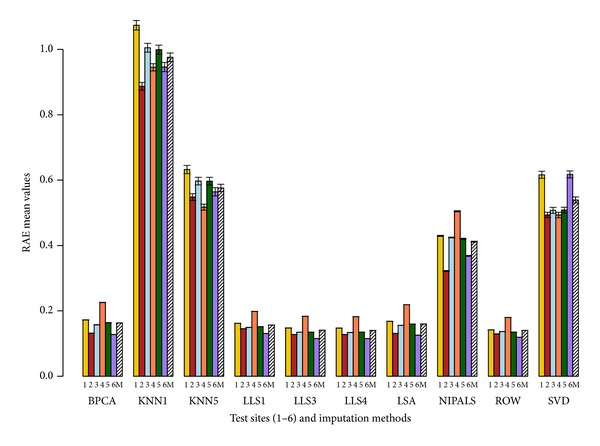
Average RAE barplot with error bars. RAE values are represented on the *y*-axis. The *x*-axis has the 6 sites (1, 2, 3, 4, 5, and 6) and 10 imputation tests (BPCA, KNN with *k* = 1,5, lls with *k* = 1,3, 4, LSA, NIPALS, ROW, and SVD). Mean (M) depicted by the slashed bar represents the overall mean for individual IM where the RAE values are averaged across the 4 pools and 6 sites. This figure shows the performance of the 10 imputation tests using the RAE metric with 5% deletion of values. 1000 simulations were performed where each simulation generated a dataset containing 5% missing values by randomly removing probe set values from the complete expression matrix of probe sets. Missing values were imputed using the 10 imputation tests. The results are compared using the RAE metric (see Section 2). The RAE values are averaged across the 4 pools. LLS with *k* = 4 has the best performance as it has the lowest RAE value for a given site. KNN with *k* = 1 has the highest RAE value and has the worst performance for all pools and all sites.

**Figure 5:**
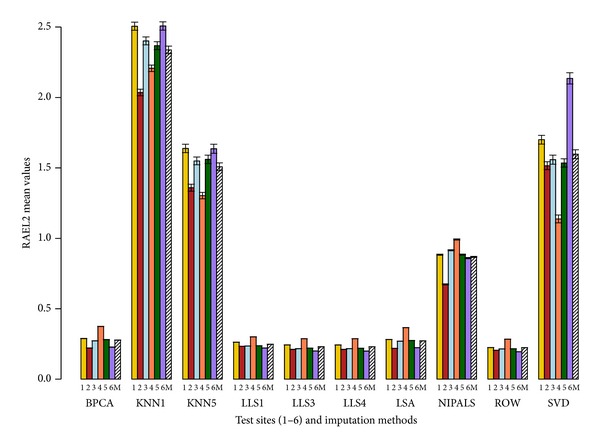
Average RAEL2 barplot with error bars. RAEL2 values are represented on the *y*-axis. The *x*-axis has the 6 sites (1, 2, 3, 4, 5, and 6) and 10 imputation tests (BPCA, KNN with *k* = 1,5, LLS with *k* = 1,3, 4, LSA, NIPALS, ROW, and SVD). Mean (M) depicted by the slashed bar represents the overall mean for individual IM where the RAEL2 values are averaged across the 4 pools and 6 sites. This figure shows the performance of the 10 imputation tests using the RAEL2 metric with 5% deletion of values. 1000 simulations were performed where each simulation generated a dataset containing 5% missing values by randomly removing probe set values from the complete expression matrix of probe sets. Missing values were imputed using the 10 imputation tests. The results are compared using the RAEL2 error measure (see Section 2). The RAEL2 values are averaged across the 4 pools. ROW has the best performance as it has the lowest RAEL2 value for a given site. KNN with *k* = 1 has the highest RAEL2 value and has the worst performance for all pools and all sites.

Figures 2, 3, 4, and 5 show the performance of different imputation methods for each error measure for all the pool and site combinations for 5% deletion. Further supplemental figures and tables showing the performance of different imputation methods on each site and pool as measured by the 4 error measures are found in Sadasiva Rao et al.'s work [27]. Results from 5% deletion and 10% deletion show a similar pattern. As expected the imputed values and variance with 10% missing data are larger than 5% missing data. Site 4 has the highest values for most of the imputation tests for all the samples (see Sadasiva Rao et al.'s work [27] for more details). Ultimately, LLS with *k* = 4 has the best performance with 10% deleted values.

## 4. Discussion

The MAQC project allows researchers to study a variety of microarray aspects including comparisons of one-color and two-color arrays [28], reproducibility [14, 15, 29], removal of batch effects [30], and determining differentially expressed genes [31]. From this diverse research, it is clear that the MAQC projects represent a fertile testing ground for microarray inspired algorithms and methods. However, to date, we are not aware of any work examining imputation methods on the MAQC datasets. 

Our conclusion is that LLS with *k* = 4 has the best performance given our set of error measures. We note that the optimality of LLS with *k* = 4 is not uniform across all error measures, sites, and pools. Also, in Figures 2–5, it is clear that several other imputation methods offer similar performance to LLS with *k* = 4, for example, LLS with *k* = 1,3, LSA, and BPCA. These results are similar to those found by Brock et al. [8] and commented on by Aittokallio [32] concluding that the top performing imputation algorithms (LS, LLS, and BPCA) are all highly competitive with each other, but no method is uniformly superior in all analyses. To that end, Brock et al. [8] develop measures to determine the appropriate (optimal) imputation method for a given dataset based on the correlation within the dataset. 

We choose a set of cutting edge imputation schemes to apply in the MAQC datasets. There are numerous applied references for the imputation schemes including [7–10, 32–34]. Optimality in the imputation schemes was assessed via (1) raw score error measures and (2) rank-based error measures taken across our cohort of error measures. The error measures chosen (see Secton 2) were designed to assess (1) errors in raw expression values (RMSE), (2) errors in the logarithm transformed expression values (LRMSE), (3) relative errors designed to penalize errors relative to the raw expression values (RAE), and (4) relative errors designed to penalize the error relative to the logarithm expression value (RAEL2). Hence, there are 2 (relative and absolute) error measures based on raw expression scores and 2 error measures (relative and absolute) based on the logarithm of expression values. Because of this balanced design in error measures between relative and absolute measures and raw and logarithm transformed data, it is reasonable to compute the average rank across these error measures to assess the overall quality of an imputation method (see Table 1). Thus, these rank-based error measures shown in Table 1 summarize the results in a straightforward manner across sites, pools, and error measures. Note that we set *ϵ* = 0.20 for the RAE error method. For future work, our group is interested in studying the robustness of RAE to the choice of *ϵ*. We also include the raw score error measures to demonstrate the best imputation methods regardless of the employed set of the imputation methods (see Figures 2–5). 

Our study is designed with the technical MAQC dataset in mind. Thus, our error measures do not include biological measures of the type discussed in [10]. These biological measures are designed to study the clustering and classification schemes commonly applied to gene expression microarrays. While our summary error measures are important to compare the imputation schemes, it is not clear how the different imputation procedures will affect downstream biological analysis and interpretation. It is outside of the scope of this paper to address this biological question since the MAQC experiment does not represent a real biological experiment. 

We study imputation methods while using the MAS 5.0 algorithm as the preprocessing method. However, there are other preprocessing algorithms such as RMA [35–37] and GCRMA [38] that are routinely used, and these methods may influence the performance of the imputation scheme. We highlight several works that extensively study and compare preprocessing schemes for Affymetrix datasets including [17, 18, 39, 40]. It is of future work to compare imputation methods across different preprocessing algorithms. 

We recognize that the MAQC datasets are not without criticism. For example, the issue of choosing an overall optimal preprocessing scheme is still an open question [41]. Another serious criticism is provided in [42] with a reply by Shi et al. [43]. In that discussion, one of the main concerns involves technical versus biological variation. This important issue has arisen when studying other “technical” microarray datasets [39]. Considering both aspects of this question, if we use datasets containing biological and technical variation, that is, datasets designed to answer biological questions, then there are biases due to the intent of the original datasets (e.g., biological variation of the species, sample preparation, procurement of RNA, and hybridization affinities). 

## 5. Conclusions

Missing values in microarray experiments are a common problem with effects on downstream analysis. Many variables such as the biological variability of the dataset, experimental conditions of the study, percentage of missing values, and type of downstream analysis performed need to be considered when choosing an imputation method. 

In our work, we use the MAQC datasets with the MAS 5.0 preprocessing scheme to compare missing data imputation schemes for Affymetrix datasets. The best and worst performing imputation schemes remain the same for both 5% and 10% deletion percentages. We observe that *k*-nearest neighbor method with *k* = 1 has the worst performance among the imputation schemes across all error measures. Local least squares (LLS) method with *k* = 4 gives the best performance for imputing missing values across all error measures for both 5% and 10% deletion. These conclusions are based on studying 10 imputation methods with 4 error metrics and 1000 Monte-Carlo simulations. 

## Figures and Tables

**Table 1:** Summary of imputation methods for 5% and 10% deletion.

Error metric	RMSE		LRMSE		RAE		RAEL2		Average	
Deletion %	5%	10%	5%	10%	5%	10%	5%	10%	5%	10%
BPCA	2.38	2.33	5.5	5.71	5.13	5.46	5.63	5.67	4.66	4.79
**KNN1**	**9.79**	**9.79**	**9.88**	**10**	**9.83**	**10**	**9.79**	**9.88**	**9.82**	**9.92**
KNN5	7.83	7.83	9.08	8.88	8.33	8.75	8.42	8.79	8.42	8.56
LLS1	5.17	5.21	4.29	4.25	4.67	4.5	4.38	4.33	4.63	4.57
LLS3	3.83	3.75	2.17	2.25	2.13	2.17	2.17	2.17	2.57	2.58
**LLS4**	**2.79**	**2.92**	**2.29**	**2.92**	**1.96**	**1.96**	**2**	**2.08**	**2.26**	**2.48**
LSA	1	1	4.88	4.92	4.33	4.5	4.71	4.71	3.73	3.78
NIPALS	7.25	7.25	7.33	6.96	7.33	7.08	7.33	7.04	7.31	7.08
ROW	6	5.96	1.5	1.5	2	2	1.58	1.46	2.77	2.72
SVD	8.96	8.96	8.29	8.08	8.29	8.17	8.33	8.29	8.47	8.38

Rows correspond to imputation methods and columns correspond to error measures with the last columns showing the average across the error measures. Each imputation method is ranked based on its average rank performance across all pools and all sites. The rank values for every error measure and imputation method combination are averaged across the 6 sites and 4 pools as detailed in Section2. Smaller average rank values suggest more accurate imputation methods. From the table, we observe that RMSE metric suggests that LSA imputation method has the best performance. With LRMSE and RAEL2 metrics, ROW is the best imputation method. LLS with *k* = 4 (LLS4) has the best performance when we use the RAE error measure. KNN with *k* = 1 (KNNl) has the highest rank value for any given error measure; thus, it is the worst performing imputation method. LLS with *k* = 4 (LLS4) has the overall best performance across the different error measures. These results hold true for both 5% and 10% deletion.
